# Poly(Dopamine) Coating on 3D-Printed Poly-Lactic-Co-Glycolic Acid/β-Tricalcium Phosphate Scaffolds for Bone Tissue Engineering

**DOI:** 10.3390/molecules24234397

**Published:** 2019-12-02

**Authors:** Zhimin Xu, Ningning Wang, Peng Liu, Yidan Sun, Yumeng Wang, Fan Fei, Shichen Zhang, Jianying Zheng, Bing Han

**Affiliations:** Department of Oral and Maxillofacial Surgery, School of Stomatology, Jilin University, Changchun 130021, China; xuzm19@mails.jlu.edu.cn (Z.X.); nnwang19@mails.jlu.edu.cn (N.W.); jdliupeng@163.com (P.L.); sunyd18@mails.jlu.edu.cn (Y.S.); ymwang18@mails.jlu.edu.cn (Y.W.); ff18043806011@163.com (F.F.); zhangsc18@mails.jlu.edu.cn (S.Z.); zhengjy18@mails.jlu.edu.cn (J.Z.)

**Keywords:** poly-(lactic-co-glycolic acid), β-tricalcium phosphate, polydopamine, 3D printing, bone tissue engineering, cranial defects

## Abstract

Bone defects caused by osteoporosis, bone malignant tumors, and trauma are very common, but there are many limiting factors in the clinical treatment of them. Bone tissue engineering is the most promising treatment and is considered to be the main strategy for bone defect repair. We prepared polydopamine-coated poly-(lactic-co-glycolic acid)/β-tricalcium phosphate composite scaffolds via 3D printing, and a series of characterization and biocompatibility tests were carried out. The results show that the mechanical properties and pore-related parameters of the composite scaffolds are not affected by the coatings, and the hydrophilicities of the surface are obviously improved. Scanning electron microscopy and micro-computed tomography display the nanoscale microporous structure of the bio-materials. Biological tests demonstrate that this modified surface can promote cell adhesion and proliferation and improve osteogenesis through the increase of polydopamine (PDA) concentrations. Mouse cranial defect experiments are conducted to further verify the conclusion that scaffolds with a higher content of PDA coatings have a better effect on the formation of new bones. In the study, the objective of repairing critical-sized defects is achieved by simply adding PDA as coatings to obtain positive results, which can suggest that this modification method with PDA has great potential.

## 1. Introduction

Owing to the complexity of three-dimensional craniofacial defects, the repair of such bone defects is challenging [1]. The goal of tissue engineering is to reconstruct the defective tissue with an excellent biocompatibility, mechanical performance, and intricate geometry, and support the cell viability, proliferation, and migration [2,3]. Many studies use conventional methods to prepare 3D scaffolds, such as polyurethane foam, pore-forming templates, freeze-drying, and solvent casting. However, the drawback of these methods lies in the difficulty associated with handling the biophysical properties, such as the pore size, inter-well connectivity, and porosity of the 3D scaffold. Disruption of the micro and macro architecture, including the deformation, contraction, or expansion of the 3D-printed substitute, makes in-body implantation and precise repair challenging [3,4,5]. With the improvement of the current 3D printing technology, the resolution of computed tomography (CT) and magnetic resonance imaging (MRI), a 3D model of the defect area can be generated using the mirroring technique. Moreover, the ability to pre-set the data input can allow the pore morphology, pore size, pore connectivity, and porosity of the 3D-printed scaffold to be controlled, which is then converted into a multi-layer printing sequence and results in layer-by-layer cumulative manufacturing [6,7,8,9]. Unlike the conventional methods for preparing bone tissue engineering scaffolds, 3D printing techniques do not require the pre-preparation of the female mold of the scaffold and have the advantage of printing curved surfaces or even more intricate geometries. Therefore, they allow for individualized defect-specific 3D printing to fit various complex defects in clinical scenarios [10].

Some studies reported the application of 3D printing techniques in preparing collagen [11,12], poly-caprolactone [13], hydroxyapatite, and tricalcium phosphate [7,8]. To date, synthetic polymer and bio-ceramic composite scaffolds have been extensively reported for their good osteo-inductivity and desirable mechanical strength. Among all of the available materials, the polylactic acid glycolic acid copolymer (PLGA)/β-tricalcium phosphate (β-TCP) composite material has become a hot research topic in bone tissue engineering, but the ordinary PLGA/β-TCP scaffold hinders its further development in bone biomaterials, owing to its poor cell adhesion and unsatisfactory osteogenesis [4,14,15]. In 2007, a simplified method for surface modification based on the mussel-inspired polydopamine (PDA) was reported by Messersmith’s group [16]. The underlining mechanism of the mussels’ adhesion to rocks in wet environments is that dihydroxyphenyl alanine (DOPA) and lysine-enriched proteins are secreted by the mussels, and this has caused great interest in this field [17,18]. Specifically, dopamine (DA) contains both the catechol and amine functional groups, making the PDA unique, as it has the ability of self-polymerization via the oxidation of DA in the weak alkaline buffer solution and can be deposited on any surface, regardless of the hydrophobic or hydrophilic condition [19,20]. The independent characteristic of PDA coating can be easily made and quickly obtained via the self-triggered oxidation and polymerization of DA, and the PDA layer provides a platform for the deposition of metal and bio-ceramic, as well as the co-valent immobilization of several serum proteins. The hydrophilicity and functional groups on the surface can improve the cell adhesion and differentiation over the PDA/polymer/calcium phosphate composition [21,22,23].

The objective of this study is to develop a simple method to improve the osteointegration of 3D-printed PLGA/β-TCP scaffolds via the addition of PDA coating. The polymer/calcium phosphate composite was incorporated into the dopamine coatings using a simple one-step coating procedure. The study analyzed the characterization and biocompatibility of 3D PLGA/β-TCP printed scaffolds, with or without PDA coatings, and compared their osteogenesis potential via in-vitro and in-vivo experiments to determine the osteogenesis potential of PDA/PLGA/β-TCP scaffolds in comparison with PLGA/β-TCP scaffolds.

## 2. Results

### 2.1. Characterization of PDA/PLGA/β-TCP Composite Scaffolds

#### 2.1.1. Structural Features of Scaffolds

**Figure 1:**
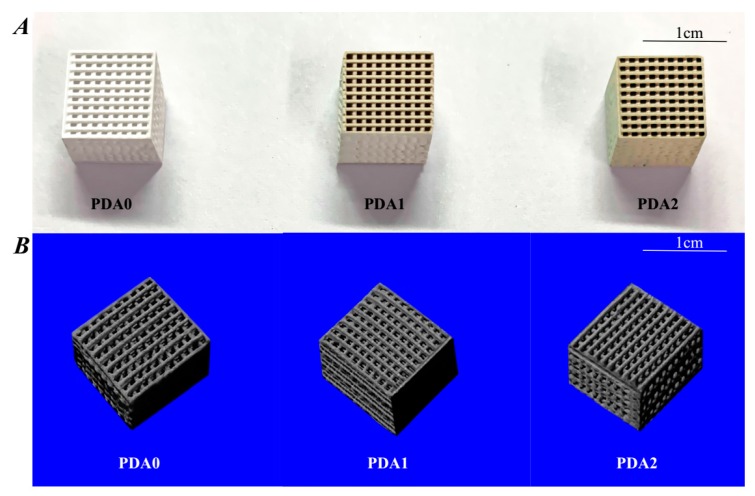
Overall appearance and structure of different scaffolds. (**A**) 3D-printed PLGA/β-TCP composite scaffolds with different PDA coatings; (**B**) reconstructed images from the micro-CT of the 3D-printed composite scaffolds with different coatings.

**Figure 2:**
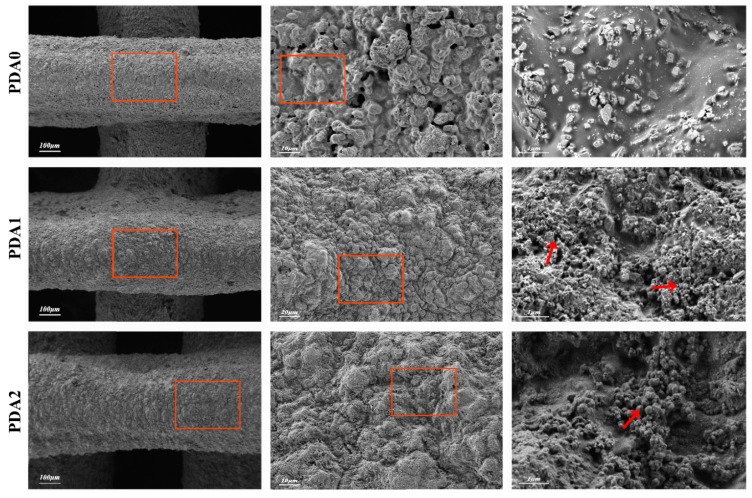
Surface morphology of different groups of scaffolds, observed under SEM. The images of different scaffolds show the surface features of the PDA0 group, PDA1 group, and PDA2 group at different magnifications. PDA is formed into a film-like coating on the surface of the scaffolds at a high magnification (red arrows).

The scaffolds’ physical appearance and micro-CT images are shown in Figure 1A. The scaffolds have a regular shape, with a uniform pore size of ~500 μm. Likewise, the 3D micro-CT images (Figure 1B) indicate a ‘honeycomb-like’ porous scaffold, which is beneficial for the in-growth of bone tissues. The printed filaments are exhibited in a network structure, with a strong architectural integrity. The surface morphology of the fiber-aligned scaffolds, with or without PDA surface modification, can be characterized by SEM (Figure 2). The results show that PDA0, PDA1, and PDA2 scaffolds have pores distributed over the internal walls, with a highly roughened structural coating layer and uneven micro-surface in comparison with non-coated scaffolds.

#### 2.1.2. Surface Wettability and Pore-Related Parameters

**Figure 3:**
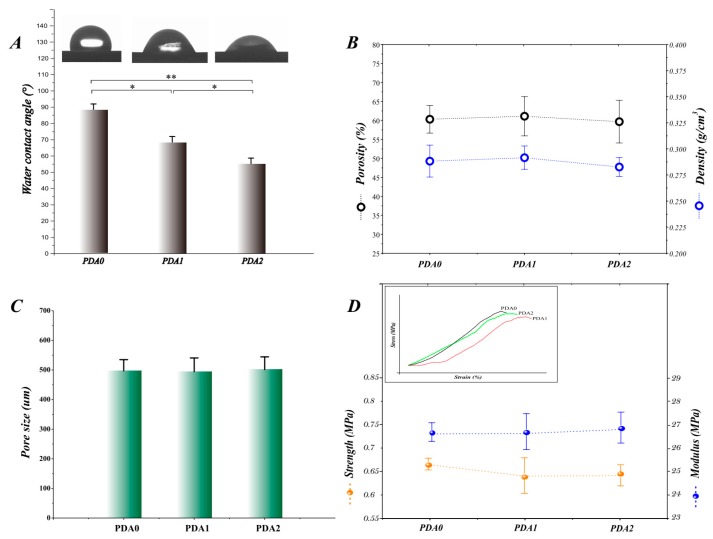
Characterization of the scaffolds from different experimental groups. (**A**) shows the water contact angle of the surface of the scaffolds of different groups; (**B**) shows the density and porosity of the scaffolds in different groups, where any differences in statistics are not indicated, and the scaffolds of different groups have a porosity of approximately 60% and density of approximately 0.28 g/cm3; (**C**) represents the average pore sizes of the scaffolds in each group, which are all approximately 500 μm, without differences; (**D**) indicates the mechanical properties, including the strength and modulus, and the affiliated figure denotes the trend of the scaffolds in each by a strain–stress curve. The results do not display any differences between the different groups, which are 0.65 MPa in strength and 26.5 MPa in modulus.

The surface wettability of PDA0, PDA1, and PDA2 scaffolds is examined using water contact angle measurement (Figure 3A). PDA0 presents the highest contact angle, followed by PDA1 and PDA2, which are 88.3°, 68.5°, and 55.1°, respectively (*p* < 0.05), and these results indicate that PDA modifications improve the surface wettability of PLGA/β-TCP scaffolds. The porosity, density, and pore size of the porous composite scaffolds are shown in Figure 3B–C. The porosities for PDA0, PDA1, and PDA2 are 60.31%, 61.1%, and 59.67%, respectively. It is worth noting that the porosities of the composite scaffolds can be kept high to meet the requirement of bone tissue engineering. On the other hand, the density fluctuates by approximately 0.288g/cm^3^, 0.292g/cm^3^, and 0.283g/cm^3^, which is already very low, with the porosity to reach the biomimicry need. PDA1 and PDA2 scaffolds have interconnected open pores, with a pore size of 449–541 μm and 458–544 μm, respectively, which is similar to the uncoated ones (460–535 μm), and all of the pores homogeneously infiltrate the 3D scaffolds.

#### 2.1.3. Mechanical Properties

Mechanical tests are performed to assess the strength of the porous scaffolds, and Figure 3D shows the results of the yield strength and compressive modulus of the scaffolds. The yield strengths for PDA0, PDA1, and PDA2 are 0.64 MPa, 0.67 MPa, and 0.67 MPa, and the compressive moduli of them are 26.2 MPa, 26.5 MPa, and 26.5 MPa, respectively. With regard to the pure PLGA/β-TCP scaffolds, the PDA coatings do not cause an apparent decrease in the mechanical properties.

### 2.2. Cell Adhesion, Toxicity, Proliferation, and Osteogenesis

#### 2.2.1. Cell Attachment

**Figure 4:**
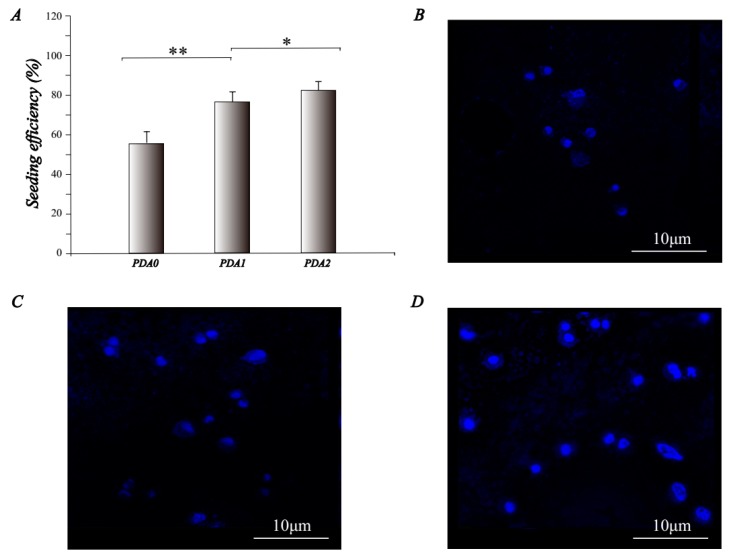
The cell-adhesion properties of the scaffold. (**A**) shows the adhesion efficiency, when cells were seeded into the scaffold; (**B**–**D**) are the pictures under a confocal laser microscope, stained by DAPI, which represent the PDA0, PDA1, and PDA2 groups, respectively.

Mouse pre-osteogenic cells (MC3T3-E1 cell line) are selected for cell attachment tests. Figure 4A shows that the cell seeding efficiencies of the PDA0, PDA1, and PDA2 scaffolds are 56%, 76%, and 82%, respectively, and the PDA2 group is the best in terms of its adhesive abilities (*p* < 0.05). The surface inoculation efficiency increases significantly with the increase of the calcium phosphate content, and the PDA2 scaffolds have a significant advantage over the other two groups (*p* < 0.05). The morphology and main distribution of the cells adhered to the scaffolds are observed by confocal laser microscopy, which is shown in Figure 4B–D. The results show that as the concentration of PDA coating increases, the cells adhering to the scaffold gradually increase in number, and the spread of the cell membranes also becomes broader with the growth of some pseudo-podiums.

#### 2.2.2. Cytotoxicity

**Figure 5:**
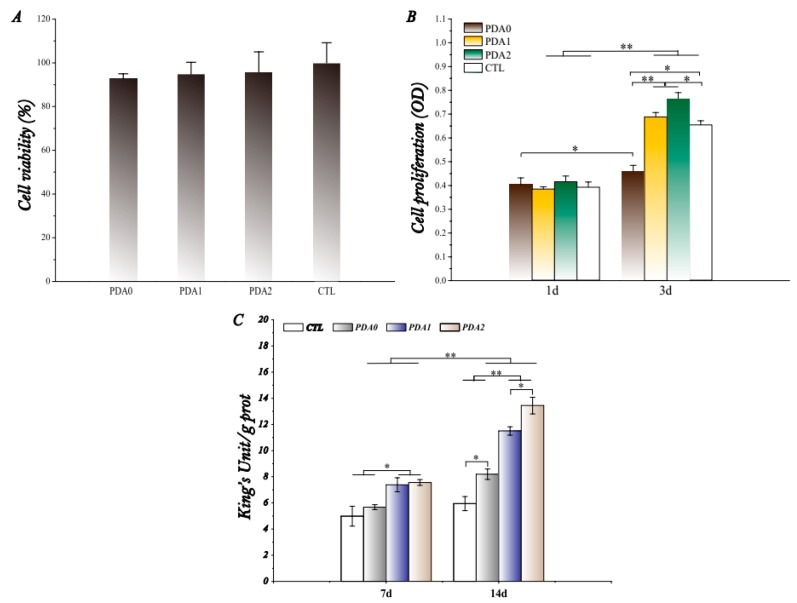
The biocompatibility test results of the scaffolds in different groups. (**A**) indicates the indirect toxicity of the extract from scaffolds to the cells, where all the scaffolds do not have cytotoxicity to the cells; (**B**) represents the proliferation activity of the cells on the surface of the scaffolds; (**C**) shows the intracellular ALP content of the cells cultured on the scaffolds, meaning the degree of differentiation of the osteoblast-like cells.

The cell cytotoxicity is detected by an MTT assay. Figure 5A shows that all the scaffolds have no significant toxic effects, compared to the no-scaffold group (positive control group; CTL), and the survival rate of each group is greater than 90%.

#### 2.2.3. Cell Proliferation

After the inoculation of cells on three different scaffolds for 1 and 3 days, the effect of the scaffold on MC3T3-E1 proliferation was examined using the CCK-8 method. The results are shown in Figure 5B. It is not difficult to see that all the scaffolds have a good biocompatibility, when comparing the results of day 1 and day 3, where the cells on all scaffold groups have excellent proliferative abilities (*p* < 0.05). On the first day, the scaffolds in the four groups do not have significant differences, but after the 3-day culturing time, the proliferation of cells on the PDA1 and PDA2 scaffolds is significantly better than that of the PDA0 group (*p* < 0.05), which is closely related to the PDA coatings. Interestingly, the cell proliferation rate for the positive control group is significantly higher, compared to the PDA0 group (*p* < 0.05), which is unexpected and may be related to the poor cell adhesion to the PDA0 surface. It also subsequently limits the cell infiltration and hinders the cell proliferation.

#### 2.2.4. Osteogenic Differentiation

The alkaline phosphatase (ALP) activity of the MC3T3-E1 cells on 3D-printed BCP scaffolds and PDA-coated scaffolds is shown in Figure 5C. The results show that the ALP activity of MC3T3-E1 significantly increases with time over the entire experimental period (7 days and 14 days) in the 3D-printed scaffolds and blank wells (*p* < 0.01). On day 7, the intracellular ALP activity of the PDA1 group and the PDA2 group is significantly higher than that of the PDA0 group and the CTL group (*p* < 0.05), indicating that the PDA-coated scaffolds have an advantage in promoting osteogenic differentiation in the early stage of osteogenic induction, but PDA1 and PDA2 scaffolds have no significant differences. On day 14, the ALP content of the cells in the PDA-coated group increases significantly in comparison to the PDA0 and CTL groups, indicating that the osteogenesis in the PDA coating groups gradually increases with time. The ALP activity of the PDA2 group is significantly higher than that of the PDA1 group (*p* < 0.05), indicating an osteogenesis advantage, with a higher PDA concentration. Nevertheless, the osteogenic differentiation results of the PDA0 group are better than those of the control group.

### 2.3. Osteogenesis In Vivo

#### 2.3.1. Bones and Reconstructed Images

**Figure 6:**
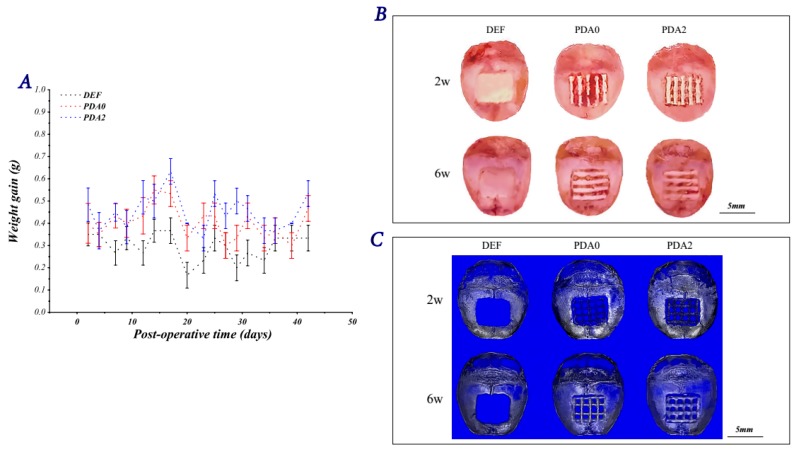
Animal experimental results. (**A**) denotes the change in body weight, after the implantation of scaffolds to the mouse skull defect. Gross specimens (**B**) and micro-CT reconstructed images (**C**) of experimental animals at 2 weeks and 6 weeks after scaffold-implantation surgery.

The body weight of the mice is monitored after scaffold implantation, and the results are displayed in Figure 6A. Figure 6A shows that the body weight of the experimental animals without the scaffolds is always lower, and this may be related to the destruction of the tissue integrity. At 2 weeks after the implantation of the scaffolds, no significant bone healing occurs in the cortical bone and internal implants in the DEF group (bone defects without scaffolds). At the same time, PDA2 scaffolds in the bone defects are fixed in place and rigid, compared to the other groups, and they are loosely in connection with surrounding tissues, which proves that there might be a strong bonding at the scaffold–host bone tissue interface in the PDA2 group. At 6 weeks post-surgery, no significant new bone formation is observed in the DEF group either. In contrast, newly formed bone tissue can be observed in the PDA0 and PDA2 groups, and the new bones are observed in the defect site at the edge of the scaffolds, with the pores of both PDA0 and PDA1 groups being smaller in comparison to the prior implantation. Overall, the changes in the PDA2 group are more prominent in comparison to those in the PDA0 group (Figure 6B). The osteogenic performance of the scaffolds in each group is further confirmed postoperatively by micro-CT analysis at 2 and 6 weeks (Figure 6C). The PDA0 and PDA2 groups show significant new bone formation in the critical-sized defects of murine skulls from week 2 to week 6, which indicates that osteogenesis does occur in both the PDA0 and PDA2 groups. The bone density is the highest, and the pores of the scaffolds are the most obliterated, in the PDA2 group, compared to the other groups.

#### 2.3.2. Histological Analysis

**Figure 7:**
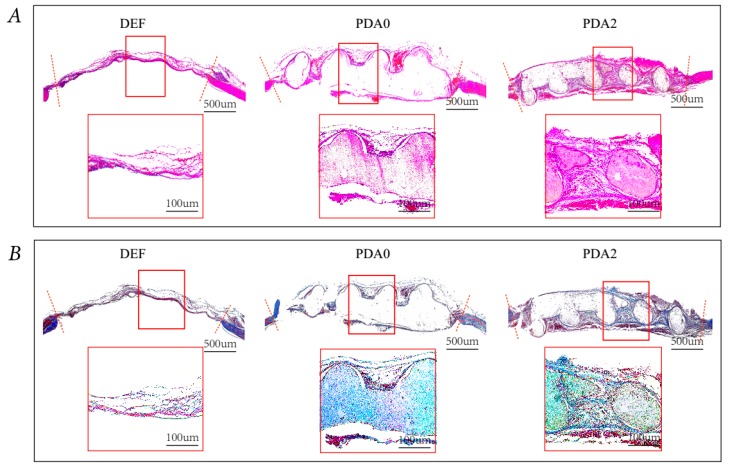
Tissue sections with the H & E and Masson staining of parietal bones from mice, 2 weeks after implantation. (**A**) shows the microscopic appearance of tissue sections treated with the HE staining technique and the representative area in the images; (**B**) shows that treated with the Masson staining technique. The tissue sections are shown.

**Figure 8:**
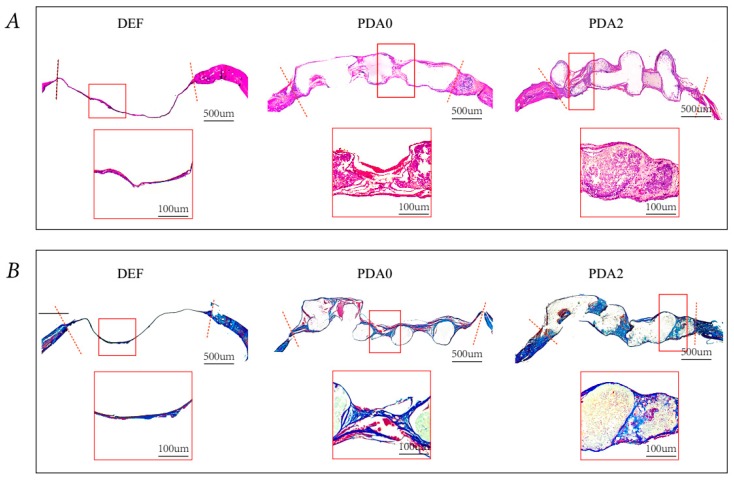
Tissue sections with the HE and Masson staining of parietal bones from mice, 6 weeks after implantation. (**A**) shows the microscopic appearance of tissue sections treated with the HE staining technique and the representative area in the images; (**B**) shows that treated with the Masson staining technique. The tissue sections are shown.

Animals were sacrificed 2 weeks (Figure 7) and 6 weeks (Figure 8) after implantation, and the bones were sliced and stained with hematoxylin-eosin (H & E) and Masson’s trichrome (MT), respectively. The fixed specimen was immersed in 10% disodium edetate (EDTA) solution for tissue decalcification for 2 weeks, prior to hematoxylin-eosin staining (HE, Figure 7) and Masson’s trichrome (MT, Figure 8B). In week 2, cells and tissues with HE were not infiltrated with inflammatory medium obviously in all the groups, but fibrous connective tissues can be observed, indicating the effective occurrence of tissue in-growth. Especially, the PDA2 group shows the bridge with a denser and thicker layer of fibrous connective tissues in comparison to the other groups, and MT staining confirms that the newly formed tissues are collagenous (blue). In week 6, a greater amount of new tissue formation along the scaffold lattices and bridge over the scaffolds in the PDA0 and PDA2 groups with mineralization can be observed, but the PDA2 scaffolds have better tissue-formatting abilities, while the DEF group shows limited amounts of formation of new tissues, as in week 2 at the defect margin. We can observe that the implanted scaffolds are rigidly fixed to the bone defects in the PDA2 group due to a match between the scaffold degradation rate and the bone repair rate. In addition, the components of calcium phosphate can partially offset the pH-reduction during the degradation of PLGA-based polymer scaffolds, because it has been proved that the slightly acidic microenvironment (pH 5.5 to 6.7) inhibits osteoblast activity and promotes bacterial proliferation.

## 3. Discussion

We successfully fabricated the tissue engineering scaffolds out of biomaterials. In terms of their appearance, the composite scaffolds act as highly roughened surfaces, and the printed filaments are even observed in homogenous pores (~500 um). Likewise, the 3-dimensional (3D) micro-CT images indicate a ‘honeycomb-like’ performance, which is beneficial for the in-growth of bone tissues [17], because the printed filaments are observed in a network structure, with a strong architectural integrity. The surface morphology of the fiber-aligned scaffolds, observed by SEM, shows that the PDA0, PDA1, and PDA2 scaffolds have pores distributed over the internal walls, and the PDA-coated scaffolds have a highly roughened structural layer and uneven micro-surface in comparison with non-coated scaffolds, which suggests that the PDA coatings can be found in the surface of scaffolds [18]. The surface wettability of PDA-coated scaffolds is better that of the uncoated ones. As is known to all, surface properties are closely related to the adhesive and proliferative abilities of osteoblasts, thus affecting the formation of new bones [24]. The hydrophilic property is very suitable for topographical environments to improve bone regeneration due to the attachment of various proteins and growth factors to these substrates, thereby enhancing the adhesion and proliferation of stem cells [20,25]. The porosity, density, and pore size results of the porous composite scaffolds indicate that the porosities can be kept high (almost greater than 60%), and although they did not reach a high porosity, like natural bones (70–80%) [18], the ~60% porosity is still good, when compared with the other 3D structural scaffolds produced using traditional methods [3,4,5]. On the other hand, the densities of the scaffolds were kept at a very low level, with a high porosity, in order to satisfy the biomimicry need [26]. The PDA1 and PDA2 scaffolds have interconnected open pores, with an average pore size of ~500 μm, which is similar to the uncoated ones (460–535 μm), and this indicates the coatings do not affect the pore-related parameters. Mechanical tests are performed to assess the strength of the porous scaffolds. With regard to the uncoated scaffolds, PDA coatings do not cause an apparent decrease in the mechanical strength, and our findings are consistent with studies that reported that PDA did not weaken the mechanical strength of scaffolds [8,27]. The hydrophilic condition is improved by the PDA coatings to promote cell activities, and they have a compact and intact porous architecture, which is predicted to improve the surface area for cell infiltration and proliferation. In addition, the multi-level pores and large specific surface area can improve the adsorption of nutrients and proteins. A strong bond and stable interconnected pores can withstand a larger load bearing [26,27,28]. These results endow the scaffold with a kind of prominent superiority, because it is lightweight, indicating that the thickness of the coatings does not have an influence on the pore size, porosity and density of the scaffolds, while the high porosity of the scaffolds can be retained.

From the cell tests, we can conclude that a high concentration of PDA coatings has the ability to improve the cell abilities in adhesion, infiltration, and proliferation. The cytotoxicity is detected by an MTT assay, and the results show that none of the scaffolds have a significant cytotoxicity, which meets the requirements of bio-safety for bio-materials. The proliferative promotion of PDA0 scaffolds is not obvious, but the PDA1 and PDA2 scaffolds are significantly in favor of cell growth, and the cells on the PDA2 scaffolds have better activities than those on PDA1 (*p* < 0.05). Based on scanning electron microscopy, the water contact angle test, and the microscopic observation, we can summarize that the water wettability plays a significant role in determining the cell compatibility and cell adhesion at the initial stage and, later, the cell proliferation (osteogenesis process). From this, we can conclude that PDA coatings can improve the hydrophilicity of scaffolds, and the concentration of PDA is proportionate to the cell proliferation, which indicates that PDA2 scaffolds are the ideal materials for bone tissue engineering [29].

PDA-coated scaffolds have the advantage of promoting osteogenic differentiation in the early stage of osteogenesis, but PDA1 and PDA2 have no significant differences, which may be explained by the fact that the osteogenic effect of PDA coatings on cells does not constitute saturation, so the differences in the groups with different PDA concentrations are not demonstrated [30]. After 14 days of culturing time, the ALP activity of all the groups increased significantly in comparison to that at 7 days, indicating that the osteogenesis in the PDA coating groups gradually increased with time. The ALP activity of the PDA2 group is significantly higher than that in the other groups, indicating the osteogenesis advantage of a high drug concentration. Nevertheless, the osteogenic differentiation results of the PDA0 group are better than those of the control group, which may be related to the internal structure of the scaffolds [31,32,33].

For the osteogenesis of scaffolds in vivo, we can observe that the implanted scaffolds are rigidly fixed to the bone defects in the PDA2 group, mainly because of a match between the scaffold degradation rate and the bony repair rate, which is very important for osteogenic repair [34,35]. In addition, the components of calcium phosphate can partially offset the pH-reduction during the degradation of PLGA-based polymer scaffolds, because the slightly acidic microenvironment (pH 5.5 to 6.7) proved to inhibit the osteoblast activity and promote bacterial proliferation, which is the main disadvantage of the osteogenic process [36,37,38]. The PDA0 and PDA2 groups show significant new bone formation in the critical-sized defects of murine skulls from week 2 to week 6, which indicates that osteogenesis does occur in both the PDA0 and PDA2 groups. Especially, the bone density is the highest, and the pores of the scaffolds are obliterated, in the PDA2 group, compared to other groups. These results indicate that the PDA-coated composite scaffolds exhibit a better osteogenic potential in the mouse skull defect model, which may be related to the inorganic ions provided by the scaffolds after degradation in vivo [39].

## 4. Materials and Methods

### 4.1. Fabrication of the PLGA/β-TCP Scaffolds

**Scheme 1:**
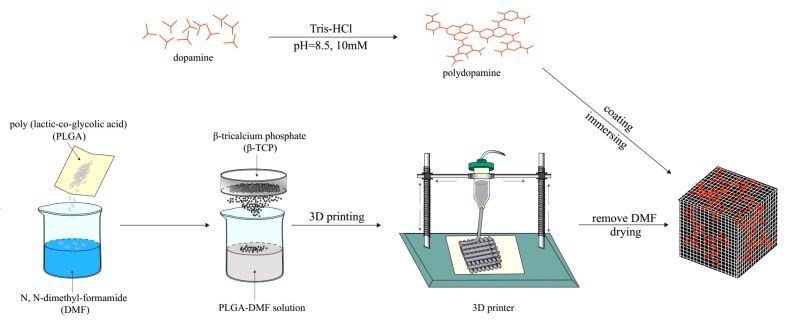
Schematic diagram of the scaffold preparation process. Dopamine (DA) is shown by the red lines, and polydopamine (PDA) is shown by the red grids. For the preparation of the printing paste, PLGA with a solid phase was put into DMF liquid, followed by adding sieved β-TCP particles, and then the composite materials were mixed thoroughly to achieve a uniform state with the inorganic particles. The prepared mixture for printing was then 3D-printed by a printer to form the design shape and structure. Finally, PDA is coated on the surface of dry β-TCP scaffolds, and the fabrication process of PDA-coated and 3D-printed β-TCP scaffolds is finished.

First, 4 g of PLGA (Mw ≈ 20000) was dissolved in 4~5 mL of DMF solvent, followed by magnetic stirring of the PLGA, until it completely dissolved. The uniform particles of β-TCP (4 g) were added to the PLGA-organic solvent solution and sonicated for 20 min. Lastly, the calcium phosphate powder was uniformly dispersed in the solution. The mixture of ultrasonically dispersed PLGA/β-TCP/organic solvent was continually stirred to form the inorganic powder particles, which avoids the clogging of the nozzle during 3D printing. The well-mixed paste was poured into the syringe of the 3D printer to fabricate the designed scaffolds, and the 3D-printed scaffold was kept in the fume hood overnight, allowing the composition of the scaffold to become solidified. Lastly, the cured PLGA/β-TCP composite material was immersed in deionized water to remove the organic solvent and dried for 3 days (Scheme 1). All stages of the operation were completed at room temperature (RT).

### 4.2. PDA Coating

An amount of 0.08 g of Tris was added to a 500 mL volumetric flask, with 400 mL of ultrapure water. The solution was kept at pH~8.5 by adding HCl. Then, 100 mL of ultrapure water was added to form the 10 mM Tris-HCl buffer solution at room temperature. Next, 100 mg and 200 mg of dopamine (DA) were dissolved in the 100 mL Tris-HCl buffer solution and centrifuged at 150 rpm at 37 °C overnight to prepare a polydopamine (PDA) solution at a concentration of 1 mg/mL and 2 mg/mL, respectively. The prepared composite scaffold was immersed in the PDA solution and centrifuged at 150 rpm overnight at 37 °C. The scaffold was then washed thrice with ultrapure water and dried overnight. The scaffold coated with 1 mg/mL of PDA solution was abbreviated as PDA1, whereas the scaffold coated with 2 mg/mL of PDA solution was abbreviated as PDA2. Lastly, PDA0 referred to the pure PLGA/β-TCP scaffold, without PDA surface modification.

### 4.3. Characterization

The pore diameter, porosity, pore connectivity, surface morphology, and microstructure of the above tissue engineering scaffolds, PDA0, PDA1, and PDA2, were observed using scanning electron microscopy (Gemini SEM500, Zeiss, Jena, Germany). The accelerating voltage was 5 kV, and the prepared scaffold was scanned and post-reconstructed by micro-computed tomography (micro CT, Skyscan 1172, Bruker, Kontich, Belgium) to observe the overall structure of the scaffold. The hydrophilic condition of the samples was characterized by measuring the water contact angles of the sample surfaces and conducted using a contact angle instrument (DSA10, Kruss, Hamburg, Germany) [30]. Briefly, the contact angles were measured using the water drop method with a water-contact-angle system, equipped with a microscope and a camera. A 2 ul droplet of distilled water was extruded from the tip of the microliter syringe to make contact with the coating surface. Images were immediately collected using the camera, and the contact angles between the drop and the coating were measured from the magnified pictures [31]. The measurement was repeated five times at five different places on each sample. The pore sizes of the composite scaffolds were calculated under the microscope using the analysis software, Nano Measurer 1.2 (Fudan University, Shanghai, China), and other pore-related parameters were tested via the liquid-displacement method. The porosity (Φ) and density (D) were calculated as follows:

(1) $$\Phi \;=\;\frac{\left[\left(Wpse-Wps\right)-\left(Wpr-Wp\right)\right]/\rho e}{Vp-\left(Wpr-Wp\right)/\rho e\;}\;\times \;100\%$$

(2) $$D=\frac{Wps-Wp}{Vp-\left(Wpr-Wp\right)/\rho e\;}$$

where *W_ps_* is the mass of the pycnometer containing dry scaffolds, and *W_pse_* is the mass of the pycnometer filled with ethanol and the scaffolds, while *W_pr_* represent the mass of residual ethanol and the pycnometer, after removing the scaffolds. *W_p_* and *V_p_* are the mass and volume of the dry pycnometer, and *ρ_e_* means the ethanol density at RH. The mechanical strength test of the scaffold was carried out using an electronic universal testing machine (Instron 1121, Instron, High Wycombe, Britain). The prepared sample was placed in the testing area of the testing machine, loaded at a speed of 0.5 mm/min, composited at 1000 N, and compressed until the test piece was deformed. The compressive moduli of the scaffolds were defined as the tangent slope of the initial liner region in the stress–strain curves. At least five specimens were tested in each test, and the results were averaged.

### 4.4. Cell Culture on Scaffolds

Osteoblast-like MC3T3-E1 cells, supplemented with Dulbecco’s Modified Eagle Medium (DMEM; Life Technologies, Carlsbad, CA, USA), containing 10% (*v*/*v*) fetal bovine serum (FBS; Gibco BRL, Carlsbad, CA, USA), were cultured under 5% CO_2_ at 37 °C, and the culture media were changed every other day throughout the experiment. Scaffolds prepared for biological experiments must be sterilized using UV irradiation and immersed in 75% ethanol aqueous solution for 2 h. Before the cytotoxicity test of the scaffolds, the extract of scaffolds had to be prepared. Briefly, the sterilized scaffolds were immersed in the medium for a period of time, the medium was collected for subsequent experiments, and the MC3T3-E1 cell line was selected as the testing cells. Next, 1 × 104 of cells were cultured in each well on 96-well plates for 24 h. Meanwhile, a quantitative volume of DMEM with FBS, immersed in PDA0, PDA1, and PDA2 scaffolds at a ratio of 0.1 g/mL, was kept in an incubator for 24 h to obtain the extract or leachate. On the second day, it can be observed that the cells had proliferated to cover more than 95% of the bottom of the culturing 96-well plates under an optical microscope, which indicates that the old medium should be placed using the obtained extracts. After another 24 h of incubation, the viability of the MC3T3-E1 cells was tested using a standard methyl-thiazoly-tetrazolium (MTT; Meilunbio, Dalian, China) assay, according to the previous documented protocol (10). After renewing the medium, 0.5 mg/mL of MTT solution was added to each sample for 4 h at 37 °C. Finally, a DMSO agent was added to dissolve the purple formazan formed in the live cells, and the absorbance wavelength of 492 nm of the resulting solution was measured by a Bio-Rad microplate reader (Iufinie M200, Tecan, Männedorf, Switzerland). The sterilized scaffolds were transferred into 96-well plates, and 2 × 103 cells in each well were cocultured with PDA0, PDA1, or PDA2 in the incubator for 1 and 3 days to investigate proliferative rate of the MC3T3-E1 cells. The number of MC3T3-E1 cells was evaluated by adding CCK-8 solution (Dōjindo Laboratories, Kumamoto, Japan) to each well, and after 1 h of incubation, a 450 nm absorbable wavelength was measured by the Bio-Rad microplate reader. The above coculture was repeated to investigate the adhesive performance of the MC3T3-E1 cells over the different scaffolds. The quantitative amounts and the spread of cells were observed under a confocal laser microscope (CLSM; LSM780, Zeiss, Jena, Germany), after staining with 4’,6-diamidino-2-phenylindole (DAPI, Solarbio, Beijing, China) for 1 h at room temperature.

### 4.5. Osteogenesis Assay

The MC3T3-E1 cell line was inoculated in the scaffold and cultured for 7 days and 14 days [26]. The medium of each well was removed and washed three times with PBS to remove unattached cells. The cells were lysed by adding 0.5% Triton-X 100 cell lysate to each well, and the sample was obtained after high-speed centrifugation. The total protein content of each well was measured using a BCA protein concentration assay kit (Solarbio, Beijing, China), and a standard curve was prepared. The OD value was measured at 562 nm using a microplate reader. The intracellular ALP content was measured using an alkaline phosphatase (ALP) kit (Beyotime, Shanghai, China), and the OD value was measured at 520 nm using a microplate reader. The ALP data were normalized by the total amount of protein, as determined by the BCA Protein Concentration Kit.

### 4.6. Implantation of Scaffolds into Critical-Sized Defects in Murine Skulls

The animal management regulations approved all protocols related to animals. BALB/c mice were anesthetized by an intraperitoneal injection of ketamine and xylazine. The hair over the parietal bones was shaved, and surface disinfection of the skin was performed. A full thickness midline incision from the nasofrontal to occipital region was made under sterile conditions. The subcutaneous tissue was dissected along the same line as the skin, and the periosteum was sharply incised and subsequently elevated off the skull to obtain sufficient exposure of bones for the dental drill. A full 4 × 5 mm defect thickness was prepared to remove the bone sheets from the middle of the dorsal calvarium with caution to prevent damage to the sagittal sinus and dura matter. Bioresorbable digitally printed PDA0, PDA1, and PDA2 scaffolds were then applied to the cranial defect (*n* = 6). Defects without an implantation of scaffolds were recorded as the DEF group. Following the procedure, Lidocaine 0.5% was applied to mice subcutaneously at the operative site, and no further analgesics were administered. A surgical wound on the skins was closed with 4-0 nylon, which was removed after 7–10 days. Water and food were given as usual, until the mice were sacrificed. Animals were sacrificed 2 and 6 weeks after surgery in a CO_2_ chamber, and the calvaria bones with scaffolds were removed, fixed and prepared for micro-computed tomography (micro-CT, Bruker, Kontich, Belgium) and histological analysis (HE and Masson Staining).

### 4.7. Statistical Analysis

A one-way ANOVA analysis was used to evaluate the significance of the differences between the means of different experimental groups. Scheffe’s multiple comparison test was used to verify the significance of the deviations of the data. In all tests, the results were considered statistically significant at a *p* value <0.05.

## 5. Conclusions

In summary, we successfully fabricated bioinspired PDA-coated PLGA/β-TCP composite scaffolds, and the prepared scaffolds were systematically characterized. The results confirm that the properties of the scaffolds are suitable to be used as bone tissue engineering scaffolds. Furthermore, the in vitro cell biocompatibility test results show that the PDA coatings can improve cell adhesion and osteogenic differentiation. To further study the osteogenic effects of the PDA-coated scaffolds, we established a mouse skull defect model and implanted the scaffolds in the defects for a period. The bones with scaffolds were extracted to be analyzed for new bone formation, and the results show that the PDA-coated composite scaffolds perform a satisfactory repair effect. In conclusion, our results demonstrate that the simplified bio-inspired surface modification of PLGA/β-TCP scaffolds by PDA is a very promising method for effectively repairing bone defects.
